# The Report of the Vivisection Commission

**Published:** 1912-03-16

**Authors:** 


### The Hospital

The Workers' Newspaper of Administrative Medicine and Institutional Life, Administration, National Insurance and Health.

No. 1340, Vol. LI. SATURDAY, MARCH 16th, 1912. PRICE ONE PENNY.

### THE REPORT OF THE VIVISECTION COMMISSION.

*Magna est veritas, et prævalebit;* although, as Huxley once said, it is surprising, considering her greatness, that truth is sometimes in no great hurry to set about prevailing. It is perhaps expecting too much to hope that the anti-vivisectors should accept as final the verdict, so entirely adverse to their aims and aspirations, just issued by the Royal Commission which has been investigating this subject for five and a half years. Nor is it in a spirit of exultation over their defeated cause that we welcome the long-delayed report; but rather of genuine rejoicing that the truth, as we in these columns have always regarded it, has triumphed so signally and that the cause of humanity is thereby furthered and assisted. The pages of the report will be unpleasant reading, we fear, for the advocates of the abolition of vivisection; for their principal champions are there laid low in a series of trenchant exposures to which every member of the Commission has adhered. Without dwelling any further on the personal discomfitures of anti-vivisectionists, let us turn rather to the positive findings of the Commission.

The kernel of the whole report is to be found in certain numbered paragraphs, which the members of the Commission regard as definitely proved and established. These conclusions are not, it is true, directly concerned with the moral and ethical questions involved; but they throw such a flood of clear light upon the practical aspects of vivisection that their influence upon public opinion is bound to be immense and far-reaching. First comes a frank recognition that certain results claimed from time to time as arising from vivisection, and alleged to be beneficial in the cure or prevention of disease, have turned out eventually to be fallacious or useless. Next it is stated authoritatively that notwithstanding such failures, much valuable knowledge has been acquired by vivisection, and that useful methods for the prevention, cure, and treatment of disease have been discovered in the same way. Further, it is shown that without vivisection this knowledge and these methods of cure could not have been obtained; and it is also stated that suffering has thereby been diminished in man *and in the lower animals*. Lastly, the Commissioners report, they believe that the further pursuit of similar methods will yield still further benefits.

These striking paragraphs convey the root of the matter so clearly that they hardly require comment or elucidation. But attention may be briefly drawn to the phrase which is italicised above—that concerning lower animals. There are those who hold that animal experimentation is unjustifiable and immoral, even though it be proved to be of benefit to the human race. Ethically we believe this view is untenable, but it is conscientiously held by many excellent citizens; and at least it is an intelligible proposition. The tender consciences of such people may possibly be less roughly pricked by the idea of legalised vivisection when they realise that animal experimentation diminishes not only human suffering but also that of animals themselves. Veterinary medicine and surgery, in fact, depend for their advancement on exactly the same methods and principles as the same sciences applied to human beings; and thus the report of the Commission will be helpful to some who have felt doubts as to man's moral right to help himself to knowledge at the expense of the brute creation.

On the moral question the Commission also listened to the conflicting views, and decided that vivisection, properly safeguarded by law (as it has been for nearly forty years) is morally justified and ought not to be prohibited. In arriving at this conclusion it is evident that two facts have weighed strongly with the Commission. One is that the evidence has convinced them of the enormous benefits which have already accrued from vivisection in the direction of diminishing pain, disease, and death; and of the practical certainty that even greater discoveries and benefits remain to be made in the same way. The other is that as long as public opinion sanctions spaying and castration, various sports of which hunting is the type, and the destruction of vermin, to prohibit vivisection would be totally unreasonable. This argument may not go far with some of the ultra-moralists; but it is one which we have often urged and it is undoubtedly, in its extensions and applications, of sufficient moment, alone and unsupported, to knock the bottom out of the entire anti-vivisectionist position.

So far, then, as those who believe in vivisection are concerned, the report of the Commission may well be left to the consideration of the intelligent public. Medical men and scientists have been attacked for years by anti-vivisectors. They have been fully vindicated by a Royal Commission; and there, so far as they are concerned, the matter ends. There, too, if the anti-vivisectionists have any discretion, they, too, will leave things; but past experience of the psychology of these people does not encourage the belief that they will do anything so sensible.

